# A Spatio-Temporally Explicit Random Encounter Model for Large-Scale Population Surveys

**DOI:** 10.1371/journal.pone.0162447

**Published:** 2016-09-09

**Authors:** Jussi Jousimo, Otso Ovaskainen

**Affiliations:** 1 Metapopulation Research Centre, Department of Biosciences, P.O. Box 65, FI-00014, University of Helsinki, Helsinki, Finland; 2 Centre for Biodiversity Dynamics, Department of Biology, Norwegian University of Science and Technology, N-7491 Trondheim, Norway; University of Missouri Kansas City, UNITED STATES

## Abstract

Random encounter models can be used to estimate population abundance from indirect data collected by non-invasive sampling methods, such as track counts or camera-trap data. The classical Formozov–Malyshev–Pereleshin (FMP) estimator converts track counts into an estimate of mean population density, assuming that data on the daily movement distances of the animals are available. We utilize generalized linear models with spatio-temporal error structures to extend the FMP estimator into a flexible Bayesian modelling approach that estimates not only total population size, but also spatio-temporal variation in population density. We also introduce a weighting scheme to estimate density on habitats that are not covered by survey transects, assuming that movement data on a subset of individuals is available. We test the performance of spatio-temporal and temporal approaches by a simulation study mimicking the Finnish winter track count survey. The results illustrate how the spatio-temporal modelling approach is able to borrow information from observations made on neighboring locations and times when estimating population density, and that spatio-temporal and temporal smoothing models can provide improved estimates of total population size compared to the FMP method.

## Introduction

Wildlife animal populations and other aspects of biodiversity are monitored for management and scientific purposes [1]. Indicators of total population size and spatial and temporal variation in density are used e.g. in assessing conservation statuses of species [2], estimating maximum sustainable yields [3] and understanding factors driving population dynamics [4]. Comprehensive counting of individuals is generally infeasible, and thus population abundance is typically estimated from a sample of direct observations or indirect signs, such as tracks, dung piles, or nests. A general problem with such data is how to convert the observations into a reliable population estimate [5].

To carry out a survey, an ecologist needs to account for the features of the focal species and the study environment. Key attributes of the species include whether the focal population is open or closed, aggregated or dispersed, rare or abundant, elusive or conspicuous, whether it is possible to identify individuals with or without marking them, and what auxiliary information is available [5]. This variation has led to a multitude of survey designs, which generally aim to optimize the reliability of the results under logistical, financial and other constraints [6].

Perhaps the simplest approach based on direct observations (visual or vocal) of unmarked individuals is to count animals from sample plots [5]. If the plots are randomly located with respect to the focal population, and if all individuals are counted from the plots, an unbiased estimate of population size can be obtained simply by dividing the observed number of individuals by the fraction of the area included in the plots. However, while perfect detection of individuals and the assumption of individuals not moving in or out of the sample plots during counting may be realistic for plants, these assumptions are rarely met with animals [5].

Distance sampling is an extension to plot sampling, where not only the number of individuals is recorded, but also their distance from the observer who is moving along a transect [7]. Statistical methods applied to such data assume that the detection probability decreases as a function of increasing distance from transect, and they can lead to additional accuracy due to the inclusion of a more realistic observation model [7].

Catch–effort sampling, often applied for fish counting, is based on comparing how much catch per effort reduces after individuals are removed from the population [5]. Capture–recapture is related to catch–effort sampling, but instead of measuring a change in catch after the removal of individuals, a number of individuals are captured, marked and released back [5]. The total population size can be estimated based on the fraction of subsequent captures that constitute of already marked individuals. The method often provides good estimates of population size, but is labor intensive and relies on the assumption of the marked individuals being representative of the entire population. The method also has the advantage of providing additional information about the individuals that were marked [5]. Camera trapping data [8] as well as genetic data based on scat samples [9] can be treated as capture–recapture data, assuming that the individuals can be identified. Surveys relying on indirect signs have the advantage that they often require smaller sampling effort than direct surveys, and that they are of a non-invasive nature [10]. As a trade-off, if not carefully accounting for the observation method, studies based on indirect signs yield estimates of relative abundance indices rather than estimates of absolute population density.

The scientist or manager interested in estimating population size faces not only the choice of the field method, but also the choice of the statistical approach needed to make valid and easily interpretable inferences from the survey data. Model-based approaches have long been popular in ecology, as they allow one to control for population processes and to account for uncertainty in observations [11], and as they can be tailored to be compatible with the chosen sampling method. More recently, models that incorporate a hierarchical structure of several processes into the same model have gained popularity [12]. Hierarchical models typically involve two main components, which account for imperfect detectability (called the observation model) and the unobservable “true” state of the population (called the process model or the state model) [13]. Detectability parameters can be estimated e.g. from replicate observations or distance based sampling [12,14]. The state model can involve parameters that account for intrinsic variation in the population processes as well as extrinsic variation in the environment, and it enables predictions over time and space [13].

Random encounter models (REM) [15] provide a model based approach for estimating population abundance from encounter rates, such as animal tracks [16], camera traps [17] or animal sounds [18], without the need of individual recognition. REM borrow concepts from the ideal gas model in physics [19], and they assume that the transect lines are randomly distributed with respect to the movements of the individuals within the focal population [20].

In this paper, we develop a spatio-temporally explicit extension of REM for converting counts of animal tracks to spatio-temporal estimates of population density. As an exemplifying case study, we consider the Finnish winter track counting (WTC) scheme, in which species are identified from snow tracks and the numbers of crossings over transect segment lines are counted [21]. In the context of the WTC, a formula to convert encounter rates to population density has been derived by several authors (e.g. [22,23,16]), and is known as the Formozov–Malyshev–Pereleshin (FMP) estimator. Let us denote by *X*_*i*_ the random variable counting the number of intersections between animal tracks of a given species and a survey transect *i*. Under very general assumptions, the expectation of *X*_*i*_ is given by

$$\text{E}\left[X_{i}\right]=\frac{2}{\pi }a_{i}\text{E}\left[L\right]M_{i}D_{i},$$ (1)

where E[*L*] is the expected distance that the animals move during one day, *M*_*i*_ is the survey transect length and *D*_*i*_ is the time the tracks have accumulated before counting (e.g. after a snow fall), typically one day in the case of the WTC. The variable of interest is *a*_*i*_, the density of animals (i.e., their number per unit area; with a unit depending on the units of the variables *L* and *D*) in the vicinity of the survey transect *i*. The expectation of total count over the survey area is then

$$\text{E}\left[X\right]=\frac{2}{\pi }a\text{E}\left[L\right]\underset{i}{\sum^{\;}}M_{i}D_{i},$$ (2)

where Σ_*i*_*M*_*i*_*D*_*i*_ is the total survey effort, *X* = Σ_*i*_*X*_*i*_ is the total number of counts, and *a* is the mean population density that is to be estimated. The FMP estimator for the mean population density is obtained by solving *a* from this equation, and by replacing the expected total number of counts by its observed value, and replacing the expected distance that the animals move in one day by an estimate of it.

Stephens et al. [16] showed by a simulation study that the FMP estimator is robust against violations of the assumptions behind Eq 2, including spatial variation in sampling effort and population density. The authors applied the method to estimate population sizes of ungulates in Russian Far East. However, the authors did not have independent data to validate the population size estimates from the empirical observations. Keeping et al. [20] continued the verification studies with simulated data and empirical data of antelopes in Botswanan savanna. Samples were collected each day by driving a vehicle along a transect and dragging a steel beam behind to erase the already counted crossings. The authors found the mean estimates to be unbiased and robust regardless of variation in animal movement patterns. The confidence intervals of FMP estimates depend on several factors such as animal track density and total survey effort [16,20]. The FMP estimator and distance sampling based estimators have been found to yield similar estimates for empirical data [20].

In this paper we extend the FMP estimator into a spatio-temporal Bayesian smoothing model that provides population density estimates for any subset of the space-time domain. The need for such an approach is motivated by the need of estimating population sizes separately for different regions, which may represent e.g. different management units, or by the need of relating variation in population size to spatially or temporally varying environmental covariates. The approach developed here allows one to estimate the population size in a given region and at a given time in a way that borrows information from the neighboring regions and times before and after the time of interest. We also consider temporal-only models that can be used to estimate a time-series of total population from data aggregated over space for cases where the spatial signal in the data is too weak to parameterize a full spatio-temporal model.

We test the performance of the method with a simulation study, in which we back-estimate known densities of simulated animal populations undergoing various scenarios of birth–death processes with various explicit movement patterns. The simulations are of a general nature and thus not meant to mimic behavior of any specific species, but as an empirical motivation, they are constructed to mimic some characteristics of the wolf (*Canis Lupus*) population in Finland. Like the FMP estimator, our method assumes that in addition to the encounter data, movement data are available for a subset of the population. As an important further extension, we also present a habitat weighting scheme that can be used to adjust for a bias originating from a non-random sampling design, such as that of the Finnish WTC, in which most samplings are done in forest land but the animals also utilize e.g. peatlands and urban areas.

## Material and Methods

We present a method that combines data from three sources to estimate population density as a function of space and time. First, encounter data such as winter track counts (WTC) provide large-scale information on population distribution. Second, GPS movement data of collared individuals provide information about daily movement distances, needed to translate counts into absolute densities. Third, combination of GPS data and habitat classification data provide information about relative uses of different habitats, which are needed to generalize e.g. the winter track counts from forests to other habitats.

We first describe the statistical methods aimed at estimating spatio-temporal population density. We then present a simulation study by which we test the performance of the method under conditions mimicking the Finnish WTC scheme.

### Statistical model for population density

We denote the focal area within which we wish to estimate the number and spatio-temporal distribution of individuals by $\Omega \subset R^{2}$. Let Λ ⊂ Ω be any subset of the focal area, and let *N*(Λ, *t*) be a random variable which counts the number of animals within Λ at year *t* ∈ {1,…,*T*}. We denote by *ρ*(***s***, *t*) the density (number of individuals per unit area) of animals around the location ***s*** ∈ Ω of year *t*. By density we mean that the expected number of animals is

$$\text{E}\left[N\left(\Lambda ,t\right)\right]=\underset{}{\int_{\Lambda }\rho \left(s,t\right)\text{d}s.}\;$$ (3)

If the animals were distributed independently of each other and each year, the number of animals *N*(Λ, *t*) would be Poisson distributed with mean given by Eq 3 [19], but in general the distribution can be over-dispersed (aggregated populations, e.g. animals moving in groups) or under-dispersed (segregated populations, e.g. territorial animals).

We assume that the density *ρ*(***s***, *t*) of the animals is of the form *ρ*(***s***, *t*) = *a*(***s***, *t*)*h*(***s***), where *a*(***s***, *t*) is a continuously varying function with respect to space and time that is essentially constant within the area of the survey transect, and *h*(***s***) measures the local effect of habitat type on density, which is assumed to be constant within each habitat type. This assumption is similar to those made in many species distribution models that account for the effect of habitat as a categorical variable (e.g. [24]). We normalize it so that *h*(***s***) = 1 if ***s*** belongs to the most common habitat type within the transects, e.g. all transects belonging to forestland in case of the WTC survey in Finland. The term *a*(***s***, *t*) accounts for large-scale variation in population density, and thus it plays a similar role as a spatio-temporal random effect does in species distribution modelling.

We are interested in the random variables *X*_*it*_ counting the number of animal tracks that cross over the survey route, where *i* indexes the location of the transect and *t* the year. Log-transforming the estimator for *X*_*it*_ (Eq 1) yields

$$\text{log}\left(\text{E}\left[X_{it}\right]\right)=\text{log}\left(\frac{2}{\pi }\right)+\text{log}\left(\text{E}\left[L\right]\right)+\text{log}\left(M_{it}\right)+\text{log}\left(D_{it}\right)+\text{log}\left(a_{it}\right)=\text{log}\left(e_{it}\right)+\text{log}\left(a_{it}\right),$$ (4)

where *a*_*it*_ is the density of animals in forestland near the survey route, and the term $log\;(e_{it})\;=\;log(\frac{2}{\pi })+log\;(E[L])+log(M_{it})+\;log\;(D_{it})$ can be considered as an offset. Here we have assumed that all survey routes are within one habitat type (e.g. in forest land in the case of Finnish WTC), and thus for the survey data it holds that *h* = 1 for which reason we have replaced *ρ*(***s***, *t*) by *a*_*it*_ in Eq 4.

As the distributional form of *X*_*it*_ depends on multiple factors which will in practice be unknown, we assume here that *X*_*it*_ follows the negative binomial distribution *X*_*it*_∼NB(*μ*_*it*_, *p*) with mean *μ*_*it*_ = E[*X*_*it*_] = *e*_*it*_*a*_*it*_, and the success probability *p* is to be estimated from the data. We modelled the spatio-temporal field *a*_*it*_ as log(*a*_*it*_) = *b*+*z*_*it*_, where the constant *b* models average population density and the term *z*_*it*_ captures spatio-temporal variation. While we will not consider such an extension here, we note that more generally *b* can be modelled as a function of covariates to study effect of biotic and abiotic factors on population density. We modelled temporal variation with the help of a first-order autoregressive (AR1) process, with parameter |*ϕ*| < 1 modelling the level of autocorrelation between subsequent years, i.e. *z*_*it*_ = *ϕz*_*i*,*t*−1_+*ϵ*_*it*_. Furthermore, we modelled the spatial structure in ***ϵ***_*t*_ with a Matérn covariance function with variance *σ*^2^ and spatial scale *κ* > 0 [25], as described in more detail in S1 Appendix.

As an alternative method, expected to be more robust if the spatial signal is too weak to be estimated, we applied temporal smoothing to the total yearly count by incorporating the negative binomial observation process and the AR1 state process into the FMP estimator as

$$\text{log}\left(\mu_{t}\right)=\text{log}(e_{t})+\text{log}\left(a_{t}\right),\;\text{log}\left(a_{t}\right)=b+z_{t},\;z_{t}=\phi z_{t-1}+\epsilon_{t},$$ (5)

where the residual is assumed to be normally distributed as *ϵ*_*t*_∼N(0, *σ*^2^) and the offset is given by ${log(e}_{t})\;=\;log(\frac{2}{\pi })+\text{log}\left(E(L)\right)+log(\sum_{i}M_{it}D_{it})$.

The models were parameterized using the R software [26] and the R-package R-INLA version 0.0–1428997066 [27] within a Bayesian framework as detailed in S1 Appendix. We used the default hyperpriors provided by R-INLA for the spatio-temporal and the temporal models. For the temporal model, we additionally considered a prior which provides less smoothing, since we found the default prior to perform poorly. This prior was specified for the transformed latent parameters (*θ*_1_, *θ*_2_) of (*σ*^2^, *ϕ*) with the prior parameters (0,1) for *θ*_1_ and (1,1) for *θ*_2_ (see [28] for further details). We used the FMP estimator (Eq 2) as a comparison benchmark for the evaluation of the spatio-temporal and the temporal models.

### Inferring movement distances and habitat use from GPS data

We assume that the movements are tracked for a representative subset of the individuals, e.g. by equipping them with GPS collars. With real data, the expected movement distance E[*L*] needed in Eqs 2, 4 and 5 could be estimated as a function of environmental and individual-specific covariates and the GPS sampling interval. For simplicity, we estimated E[*L*] here by averaging the observed GPS distances within 24 hours over the counting period and over the collared individuals.

The Finnish WTC transects are located almost exclusively in forests, but the animals also utilize other habitats such as cultivated fields, peat lands and frozen lakes and rivers. To estimate the preference or avoidance of other habitats relative to the nominal habitat type, we applied a step selection function approach [29] to the GPS data. We note that we used a step selection approach (in which only the immediate neighborhood of the present location is considered as the reference set) rather than a habitat selection approach (where e.g. the entire study area could be considered as the reference set) to avoid confounding the effect of *h* with large scale variation in population density, which is influenced also by other factors than local habitat selection and which is captured in our model by the parameter *a* (Eq 4).

When simulating the Finnish WTC survey, we used the CORINE land cover data with habitat types classified into *H* = 5 categories as described in section *Generation of simulated data*, indexed by *i* = 1,…,*H*. We divided the movement data into bursts indexed by *k* = 1,…,*K*, where one burst includes the GPS data from one individual during the WTC period (January–February of a given year). We denote by *p*_*ijk*_ an indicator function with value 1 if the individual used habitat type *i* at step *j* = 1, …, *J*_*k*_ of burst *k*, and with value 0 otherwise. We followed [30] by comparing the actual habitat use with a null expectation obtained by randomizing a set of possible null locations for each realized movement step. We defined *q*_*ijk*_ as the fraction of those null locations that fall into the habitat type *i* to which the animal could have moved in step *j* if it would not have any preference concerning the habitat type. We randomized 30 null movement steps based on the distributions of turning angles and step lengths observed for the burst *k*. For each burst, the proportions of actual and expected usages are given by $p_{ik}\;=\;{J_{k}}^{-1}\sum_{j\;=\;1}^{J_{k}}p_{ijk}$ and $q_{ik}\;=\;{J_{k}}^{-1}\sum_{j\;=\;1}^{J_{k}}q_{ijk}$, respectively. Thus, the ratio *w*_*ik*_ = *p*_*ik*_⁄*q*_*ik*_ describes the burst-specific relative (compared to the null expectation) preference for each habitat type, defined for habitat types that are available at the vicinity of the burst so that *q*_*ik*_ > 0. In this study, we simply used preferences averaged over the individuals and years, computed as

$$w_{i}=\frac{{\sum^{\;}}_{k=1}^{K}p_{ik}}{{\sum^{\;}}_{k=1}^{K}q_{ik}}.$$

We converted the preferences into weights relative to forest by

$$h_{i}=\frac{w_{i}}{w_{\text{forest}}}.$$ (6)

Given the distribution of habitats over the study area, the habitat-specific weights *h*_*i*_ can be converted into a map *h*(***s***) that gives the habitat preference for any location ***s***.

### Constructing a spatio-temporal estimate of population density

Given the spatio-temporal field *a*(***s***, *t*) and the habitat preference map *h*(***s***), the expected density of individuals is given by *ρ*(***s***, *t*) = *a*(***s***, *t*)*h*(***s***), and thus the expected number of individuals within any subdomain Λ can be computed from Eq 3. The numerical implementation of Eq 3 is described in S2 Appendix.

### Generation of simulated data

We generated simulated data that mimic the sampling design of the Finnish WTC survey [21]. This design consists of 1610 unique survey routes covering almost the whole of Finland, with an average of 494 and standard deviation of 161 routes counted during January–February annually since 1989. The transects are 12 km long equilateral triangles with each side 4 km and which are located entirely or almost entirely on forestland. The time over which the animal crossings are allowed to accumulate (e.g. after snowfall) varies between 1–3 days. In our simulations we assumed for simplicity that the accumulation period is 24 hours (we use the unit of day, so *D*_*it*_ = 1 for all *i*, *t*).

To incorporate habitat information in the simulations, we used the CORINE land cover grid of 25 m × 25 m resolution in year 2006 [31]. We used the level 1 classification of the types provided by CORINE: artificial surfaces, agricultural areas, forests and seminatural areas, wetlands and water bodies. The animal movements were bounded by the Finnish border, which was retrieved from http://gadm.org/ as a polygon and simplified by thinning the vertices.

We generated WTC data for the time period of *T* = 20 years. We used an individual-based model, in which movements of the individuals were simulated at Δ*t* = 4 hour intervals. We assumed that the population consisted initially of 200 individuals thus that its density was 6.0 × 10^−4^ km^−2^, as the area of the simulation domain (entire Finland) is 332,176 km^2^. We generated temporal variation to population size with a birth–death process as described in more detail below. The initial population size is comparable to the number of wolves (*Canis lupus*) in Finland in the 2000s [32].

To examine how the performance of the statistical framework depends on the nature of the data, we considered the following scenarios.

#### Scenario A,correlated random walk in a homogeneous landscape

In this scenario we assume a homogeneous landscape and that the initial distribution of the individuals follows complete spatial randomness. We simulated the movements assuming a correlated random walk, in which the turning angles followed the wrapped normal distribution with correlation 0.70, and the movement distances (km) followed the Weibull distribution with shape = 2 and scale = 2 providing mean ± standard deviation of *L* = 10.6 ± 5.6 km/day.

#### Scenario B, biased correlated random walk

This scenario differs from Scenario A in that we assume that each year 90% of the individuals in the population follow territorial movements, in which case we biased the random walk by 30% towards the territory center (= its original location that year) if the individual was further than 10 km from the territory center [33]. The remaining 10% of the population was assumed to consist of dispersers which follow the unbiased correlated random walk of Scenario A.

#### Scenario C, individuals move in groups

This Scenario is the same as A except that we consider each agent in the simulation as a group of individuals rather than a single individual. To do so, we randomized group size *N*_*i*_ for each simulated agent *i* from *N*_*i*_ = *n*_*i*_ + 1, where *n*_*i*_ is a Poisson distributed random variable with mean 4. In this case we started the simulation with 40 groups to have on average a total of 200 individuals also in this case.

#### Scenario D, spatial variation in the initial distribution of individuals

This scenario differs from Scenario A in that we assume a non-homogeneous initial distribution of the individuals. To do so, we generated a random Matérn field *F*(***s***) with spatial scale 100 km and variance 16, and simulated the initial locations of the 200 individuals by assuming that the initial distribution is Poisson with intensity proportional to exp(*F*).

#### Scenario E, heterogeneous landscape

Here we utilized the distribution of different CORINE habitat types within Finland. We set the habitat preferences (relative to forests) to 0.50 for peat-lands, 0.10 for fields and urban and 0.05 for water (i.e. areas covered by ice during the WTC period), and distributed the individuals initially according to these densities. The weights chosen here do not reflect any particular species, but are only to test the weighting method. At each movement step *i*, we randomized 10 candidate locations according to Scenario A, and selected the next step using the habitat preferences as randomization weights.

#### Scenario F, combined effects of Scenarios B–E

Here we assume that the individuals moved in groups rather than singly, that 90% of the groups performed territorial behavior, that the habitat preferences influence both the initial distribution and the movements of agents, and that there is additionally spatial random variation in the initial distribution of the agents.

In all scenarios, we assumed reflecting boundary conditions so that movements that would cross the Finnish border were discarded, and new candidate locations were randomized until the first inside the border was found.

To generate temporal variation in population size, we simulated a birth–death process at the beginning of each year by replacing each agent with a Poisson distributed (with mean *μ*) number of agents. If the outcome of the Poisson distribution was zero, the agent died, if it was one, the agent survived, and if it was two or more, the agent reproduced. In Scenarios C and F the entire group was assumed to die, survive, or produce a new group, the size of which was randomized as described above. To generate temporal variation in population size beyond that generated by demographic stochasticity, we simulated environmental stochasticity by sampling the expectation *μ* for each year from the log-normal distribution with the mean and standard deviation of the underlying normal distribution set to 0 and 0.1, respectively, so that the expected population size remained constant over the years, and in 95% of the years the population-level growth rate was within *μ* ∈ [0.82, 1.22].

To follow the design of the Finnish WTC survey with much missing data, we assumed that animal tracks were counted annually on 500 WTC triangles for a period of 20 years. We randomized the locations of surveyed WTC triangles among all the 1610 triangles independently for each year. In Scenarios A–D we used the real locations of the WTC triangles but randomized their rotation angles as data on those were not available. In Scenarios E and F we also randomized the locations of the triangles within the study region to ensure that at least 90% of each transect lay in forest. This resulted in coverage of 94% on forestland and 6% on the rest of the habitats. Sampling effort was corrected for transects lying partially outside the study area and on non-forestland. The latter was achieved by measuring coverage of each triangle on each habitat type and weighting those lengths with the habitat weights obtained in section *Inferring movement distances and habitat use from GPS data*.

We selected for each triangle the counting day randomly among the WTC period (first 60 days of the year), and let the crossings accumulate for 24 hours. The data thus consists of a 20-year time series of animal counts on 500 WTC triangles. Additionally, we assumed that each year 10 randomly chosen individuals were equipped with GPS collars. The receivers were assumed to be on for the entire WTC period of 60 days, with sampling frequency set to 4 hours, which equals the simulation step duration. We generated 50 replicate data sets for each of the six scenarios.

### Assessing the performance of the statistical framework with simulated data

To assess how well the statistical framework was able to estimate the total population size, we compared true population sizes to the estimated ones. To simplify the interpretation of the results, we assumed that we knew a priori in which cases the habitat composition influenced movements, and thus did not estimate the habitat preferences from the simulated movement data in Scenarios A–D.

We derived the population size estimates ${\hat{N}}_{tr}$ for year *t* and replicate *r* based on the spatio-temporal model, the temporal models with the default and alternative priors, and the FMP estimator, which approaches we abbreviate with ST, T1, T2 and FMP respectively. We compared the estimates to the corresponding true population sizes *N*_*tr*_ by the log-transformed ratio

$$\alpha_{tr}=\text{log}\frac{{\hat{N}}_{tr}+1}{N_{tr}+1}.$$ (7)

To assess if the estimates were on average over- or underestimates, we computed for each estimation method and scenario the mean *α* of the *α*_*tr*_ over the replicates and years, and its standard error. Second, to examine how well the variation in the estimated population sizes reflected the variation in the true population sizes, we regressed the log-transformed estimated population sizes against log-transformed true population sizes. We computed the proportion of variance *R*^2^ explained by the linear model, as well as its slope *β*. For completely accurate estimates, it holds that *α* = 0, *R*^2^ = 1 and *β* = 1.

To assess the validity of credible intervals (CrI) of total population size, we computed the proportion of estimates for which the true value fell within the 50% or 95% credible interval. The technical details of computing the CrI are given in S2 Appendix. For the FMP, we followed [34] by computing the 95% and the 50% adjusted bootstrap percentile confidence intervals using 1000 resamples. The proportions of true values falling within these intervals were determined by repeating the estimation of the confidence intervals 1000 times.

To assess how well the statistical framework was able to capture spatial variation in population density, we decomposed the computational domain of Finland into a regular grid of 12 × 6 cells, each being 100 x 100 km^2^ in size. We computed for each cell the true population size for each year based on the locations of the individuals in the middle of the WTC period (day 30), as well as the estimated population size based on the spatial integration carried over the focal grid cell as described in S2 Appendix. We computed the Spearman’s rank correlation between the predicted and true values for each year. To examine how the ability of the model to replicate the spatial distribution of the individuals depends on population size, we plotted the Spearman’s rank correlations against the true population size.

With limited data, the parameterization of the spatio-temporal model sometimes failed due to INLA reporting convergence problems or providing unfeasible estimates. We classified the estimation as failed if INLA reported convergence problems, provided infinite density estimates, or if the total population size was estimated to be under 1 or over 1000 individuals. We recorded the fraction of such estimates, and then removed them from the performance analysis.

## Results

### An illustration of simulated data

**Fig 1:**
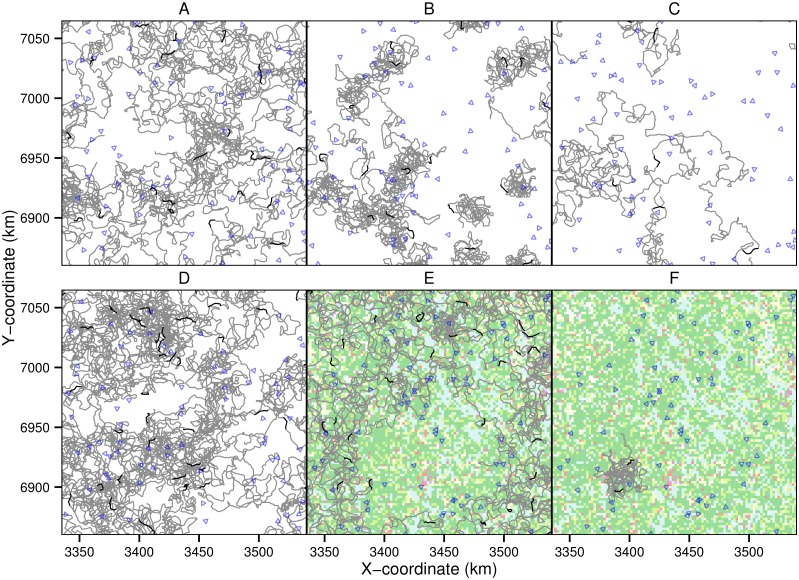
Illustration of simulated data generated by Scenarios A–F. The panels show a snapshot of movement tracks of the agents simulated for 60 days (gray lines) with the movements during counting day highlighted (black lines). The blue triangles show the survey transects. The background maps in Scenarios E–F show the different habitat types influencing movement behavior of the agents. For a description of the Scenarios A-F, see text.

A snapshot of simulated tracks, randomized transects and distributions of habitat types in the simulated Scenarios (A–F) are illustrated in Fig 1. Clustered tracks occur in Scenarios B and F due to the territorial movements, and in D and F due to the clustered initial locations. The track density is lower in Scenarios C and F as the shown tracks involve the movements of several individuals that move in a group. In Scenarios E and F, the movements are influenced by the landscape configuration (shown by the colors) due to the assumed habitat preferences of the agents.

**Fig 2:**
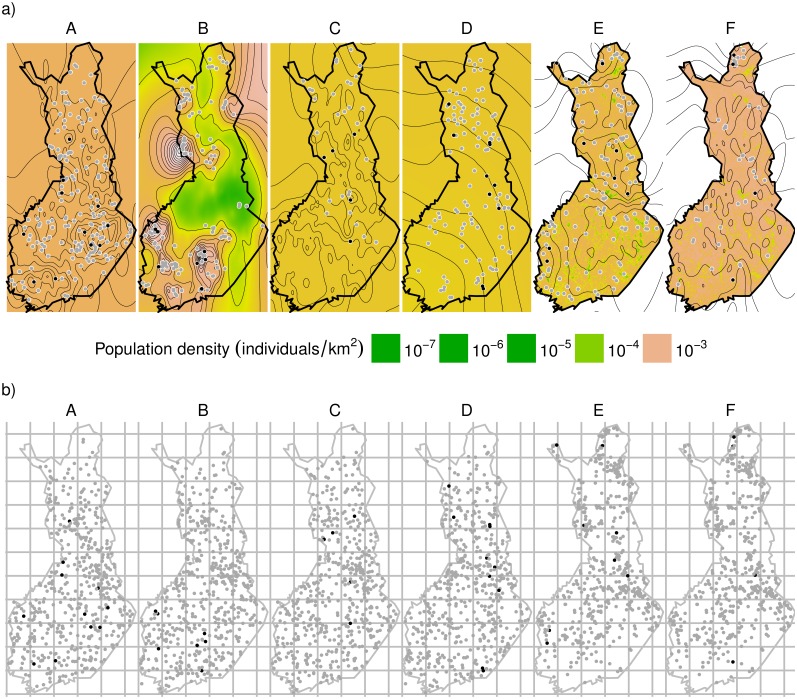
Illustration of density maps generated by the spatio-temporal (ST) model fitted to data generated by Scenarios A–F. (a) True locations of the simulated non-observed agents (grey dots), observed agents (black dots), and estimated densities from the ST model (contour lines with scale specific to each Scenario and colors with common scale). Densities in Scenarios E–F have been weighted with the habitat preferences. (b) A snapshot of survey transect locations without observations (grey dots) and with one or more observations (black dots). The rectangular grid is used to assess the ability of the spatio-temporal model to capture spatial variation (Fig 5). The data are from the 30^th^ day of the 60-day long survey period on the 10^th^ year of the 20 years. For a description of the ST model and the Scenarios A-F, see text.

Fig 2a exemplifies the large-scale distributions of the agents in the 10^th^ year of the 20 year simulation. The density surface estimates shown in this figure were obtained with the spatio-temporal (ST) model. The contour lines (which are specific to each Scenario as opposed to the common color scale) show that there is considerable spatial variation within all Scenarios. This is the case in particular in Scenario B, where territorial movements have created clustered subpopulations, and in Scenario D, where there is a density gradient from north to south due to large-scale variation assumed in the initial condition.

### Model performance in estimating total population size

**Fig 3:**
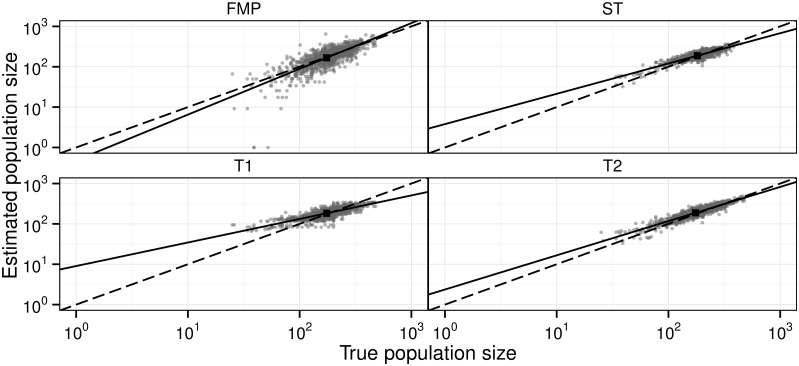
Evaluating the performance of different models in estimating total population size. The panels show the true and estimated population sizes for each year and replicate (gray dots), their means (black squares), fitted regression lines (solid black), and identity lines that would correspond to ideal fits (dashed black lines). The models are the Formozov–Malyshev–Pereleshin estimator (FMP), and the spatio-temporal (ST) and temporal (T; with priors 1 and 2) models. Note the log-scale. The figure is shown for Scenario A. Similar figures all Scenarios are shown in S1–S4 Figs.

**Fig 4:**
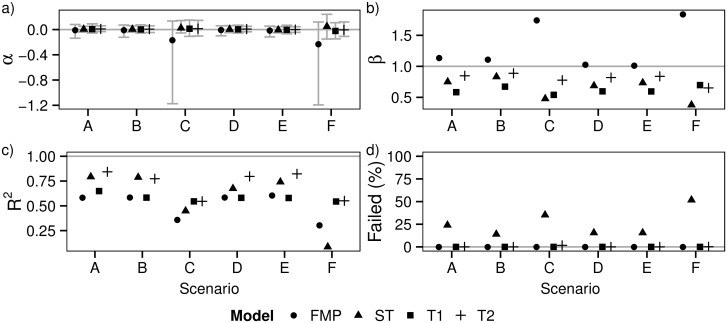
The performance of different models in estimating total population size. The panel (a) shows the means and 95% quantiles for the log-transformed ratio of estimated population size + 1 and true population size + 1 (*α*, Eq 7). The panels (b) and (c) show respectively the slope (*β*) and the variance explained (*R*^2^) in the regressions of estimated versus true population sizes (illustrated in Fig 3 for Scenario A). The panel (d) shows the percentage of estimates considered technically not valid (Failed) due to convergence issues during the estimation of the parameters. The different models (FMP, ST, T1, T2) are shown by different symbols, and the data are shown for Scenarios A–F. Numerical values are given in S1 Table.

Fig 3 compares the FMP model to the new stochastic models (ST, T1, T2) by the estimated and the true population sizes in Scenario A. As the means (marked with black squares) fall close to the identity lines, the estimates are on average unbiased, corresponding to *α* = 0 (Eq 7) in Fig 4a, and there is no difference between the models. As the slopes in Fig 3 are positive, all methods were able to capture at least some of the variation in total population size. The ST and T2 models perform the best in the sense that they explain the largest fraction *R*^2^ of variance in estimated versus total population sizes (Fig 4c, scenario A), whereas the FMP and T2 models perform the best in the sense their regression slopes *β* are closest to unity (Fig 4b, scenario A).

A comparison across the scenarios shows that the extended methods provide on average essentially unbiased estimates (Fig 4a). The FMP estimator performs poorer in cases where animals move in groups (Scenarios C and F), for which cases in a large fraction of replicates no individuals were detected at all. Sparse data make these Scenarios challenging not only for the FMP but for all methods. This is also reflected by the 95% quantiles for *α* (Fig 4a), where all estimates, and in particular the FMP estimates, have more variance for animals moving in groups. The T2 model was generally most robust in the sense of providing always high *R*^*2*^ values and *β* values close to unity, whereas the performance of the other methods was generally poorer or varied more among the scenarios (Fig 4b and 4c). The ST model performed essentially as well as the T2 model for cases where the individuals moved singly, but it performed worse for those cases where the animals moved in groups and thus the data were spatially sparse. A technical problem with applying the ST model was that INLA failed to converge frequently, in particular with sparse data (Fig 4d).

The fact that the slope *β* is less than one for all of the stochastic models is due to the AR1 component in the statistical model that results in smoothing of the data over the survey years. While such smoothing improves the models performance in some aspects, it necessarily leads to underestimation of population size in peak years and to overestimation of population size at years of low abundance. The level of smoothing depends on the hyperpriors for *ϕ* and *σ*^2^ and the initial values supplied to INLA. In case of the scenarios simulated here, the default priors and initial values for T1 resulted in over-smoothing by shrinking variance towards zero, whereas the more relaxed priors for T2 provided a much better fit (Fig 4 and S1–S4 Figs). Consistently with the fact that the FMP estimator can be considered as the limit of no smoothing at all, the slope *β* for this method is very close to unity for those Scenarios where the individuals move individually. Moreover, the FMP estimator did not suffer from technical problems. However, the trade-off of not applying smoothing is that the FMP method performs poorly in capturing variation in population size among the years (in the sense of the *R*^2^ values being low). This is the case because the FMP estimator does not utilize information from years before or after the focal year, and thus it involves more noise than the other estimates.

We note that the FMP model and the extended methods estimated the total population size in an essentially unbiased way also in the Scenarios E and F where the individuals preferred some habitats over the others, even if all sampling was conducted in the forests (Fig 4a, S1 Table). This is thanks to the habitat weights estimated from the GPS data. We repeated the estimation procedure without habitat weighting, in which case the population sizes were estimated approximately 24% higher than their true values.

**Fig 5:**
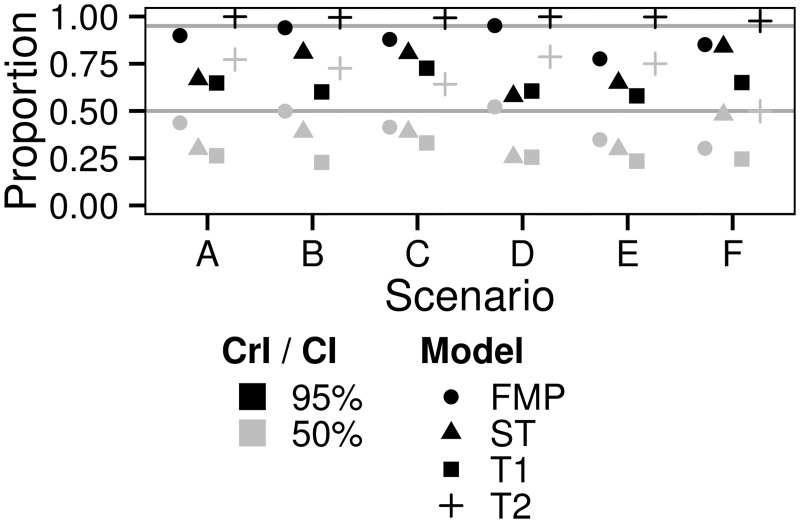
The validity of interval estimates for total population size. The symbols show the proportions of true population sizes falling within 95% (black symbols) and 50% (gray symbols) credible intervals (CrI) for the Bayesian models (spatio-temporal ST; temporal with default prior T1; temporal with custom prior T2) and confidence intervals (CI) for the FMP model in the Scenarios A–F. Ideal proportions are marked with grey horizontal lines at 0.95 and 0.50.

The credible interval estimates were somewhat biased for the FMP and all the extended methods considered (Fig 5). As the ST and T1 models suffered from over-smoothing, a high proportion of the true population sizes fell outside the credible interval. In contrast, the T2 model provided much wider credible interval that contained the true population size even more often than the nominal value of the credible interval suggested. This shows that the credible intervals are sensitive to the choice of the prior and thus that their reliable estimation with real data is challenging. The confidence intervals of the FMP model were in most cases more accurate than the credible interval estimates for the Bayesian models. Variation is expected due to the low number of simulation replicates.

### Model performance in estimating spatial variation in population size

**Fig 6:**
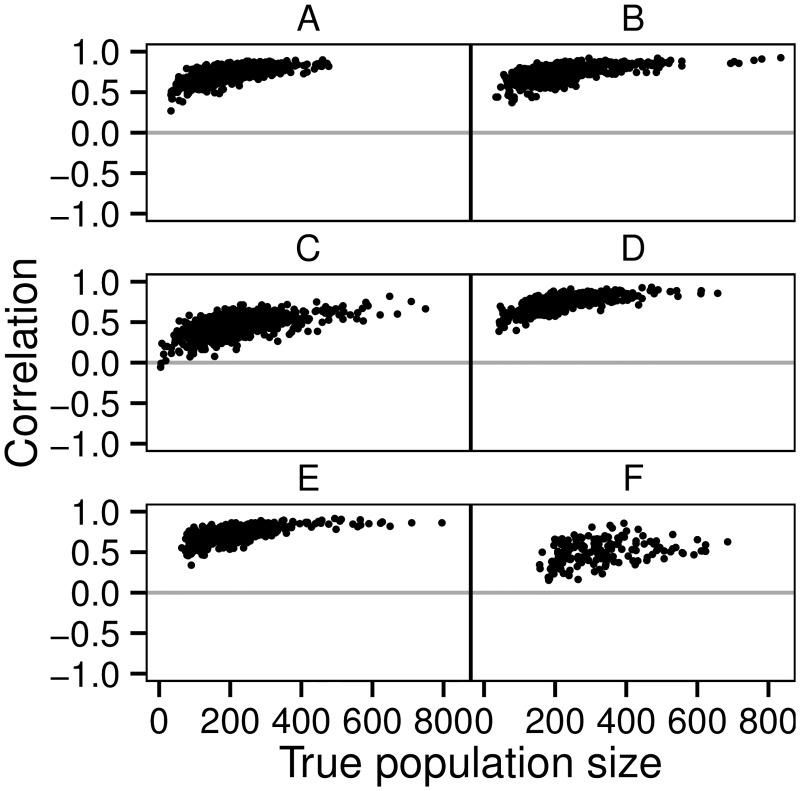
The performance of the spatio-temporal (ST) model in estimating spatial variation in population size. The dots show Pearson’s rank correlation between the true and estimated population sizes, computed for each year and each replicate from the regular grid shown in Fig 2b. The gray horizontal lines indicate no correlation. The panels correspond to the Scenarios A–F.

The ST model was generally able to capture spatial variation in population density, as illustrated by the generally positive correlation coefficients in Fig 6. Note that this is the case even in Scenarios A, C and E in which the individuals appear to be distributed relatively randomly over the study area (Fig 2). The model’s ability to capture spatial variation increases with sample size in the sense that it increases with population density and is weaker in the case of the Scenarios C and F in which the individuals are more clustered (Fig 6). Fig 2 illustrates that the ST model is able to predict high population densities also on areas where only unobserved individuals are present (grey dots on Fig 2a), demonstrating its ability to interpolate over space and time. While some individuals may go undetected in some years, the model predicts their existence based on other observations made in proximate locations and proximate years.

## Discussion

In this paper we have extended the classical FMP estimator into a temporally or spatio-temporally explicit random encounter model. We have also shown how a combination of movement and habitat data can be utilized to account for variation in population density among different habitat types, including habitats that are not sampled by the encounter data.

The main benefit of the spatio-temporal extension of the FMP model is that it provides information about spatio-temporal variation in population density in addition to a global estimate of population size. Further, the smoothing applied by the temporal and spatio-temporal methods improves their accuracy in capturing variation in population size among years, and the weighting scheme over the habitat types enables the estimation of absolute population sizes even if the sampling itself is biased to some habitat types only. These methodological improvements are essential in ecological applications, conservation and management. For example, large-scale and long-term WTC surveys have been conducted in Russia [35] and in Finland [21], but the spatio-temporal aspects of these data have thus far remained underused, and the derived estimates of population density have in most cases been considered as relative rather than as absolute (e.g. [36]).

To assess the performances of the models, we conducted extensive simulation studies, in which we included both small-scale and large-scale variation in the aggregation of the individuals, as well as temporal variation in the parameters of the underlying birth–death process. Our results confirm the earlier finding that the FMP estimator is essentially unbiased [16,20], and they show that the spatio-temporal and temporal models yield essentially unbiased results as well. What is less robust and would require more future work both with the FMP estimator and with the spatio-temporal and temporal models is the estimation of credible and confidence intervals.

If one is interested in estimating the average population density only, the simple FMP estimator even outperformed the extended models in many cases, and it can be considered as the preferred choice also due to its computational simplicity. In contrast to the FMP estimator, which assumes that all observations are independent, the extensions developed here incorporate a neighborhood structure between the observations in space and time. This generates a smoothing of the data, which reduces noise and thus reduces variance in population size estimates. Most importantly, the smoothing of the data yields not only an estimate of total population size but also an estimate of spatiotemporally varying population density. This enables interpolation, i.e. the prediction of missing data in areas and time where no surveys were conducted, as well as estimating the size of a subpopulation inhabiting any part of the domain. While the spatio-temporal model assesses variation in population density over the space-time domain, the temporal model provides only a temporal smoothing, which, depending on the application, may be sufficient. The data requirements needed for the successful application of the estimation methods increase with the complexity of the model to be fitted. In particular, the estimation of the spatio-temporal model is challenging with sparse data, with the computational algorithm behind INLA failing to converge with a substantial proportion of the cases considered here. In such a case, one has to resort to the temporal model or the FMP model. Even in cases where the fitting of the spatio-temporal model is technically feasible and where one is particularly interested in the spatio-temporal aspects of the data, we recommend fitting all model types, including FMP. This is because a comparison among them is informative about the robustness and reliability of the results.

The Bayesian approach considered here allows one to utilize prior information. As we have shown by comparing the results obtained for two different priors for the temporal model, the choice of the prior can significantly influence the results, especially if working with sparse data. This can be considered as a positive aspect, as providing an informative prior can increase the estimation accuracy. Furthermore, informative priors can enable analysis of sparse data, which kind of data have been considered problematic for WTC studies [16]. With sparse data the parameterization of the spatio-temporal model failed technically for a considerable proportion of the simulations, which problem could have been addressed by providing a more informative prior. However, the sensitivity of the results to the prior can also be considered as a negative aspect, as it means that the results can be biased by a poor choice of the prior in cases where there is no additional information about the spatial and temporal scale of population variability. For example, with our simulated case studies the default priors of INLA led to over-smoothed estimates. While with a simulation study the prior could be optimized to maximize a particular aspect of the performance of the estimation method, with empirical data such optimization is obviously not possible. Thus, without additional information, one cannot distinguish if the model is over- or under-smoothed, and if the credible intervals are optimistic or conservative. Therefore, we suggest that all the three methods presented here, as well as alternative priors for the Bayesian methods, should be used in parallel to examine the robustness of the estimates. A further way to examine the reliability of the results may be obtained by a visual evaluation of the fitted spatio-temporal fields (such as shown in Fig 2a) by experts who have field knowledge on the patterns of spatio-temporal variation on population density.

As we have taken here a model based approach to the analysis of encounter data, it is straightforward to incorporate many kinds of features in the analyses. As one example, we have introduced here a weighting scheme that can be used to adjust the predicted spatio-temporal densities for the habitat preferences of the species. If the animals show habitat preferences but they are not accounted for in the model, the density estimates will be biased. We thus recommend one to always estimate habitat preferences, and to include them in the model if they appear to be statistically and/or biologically significant. As another example, the error distribution of the model can be selected according to the nature of the data and the survey method used. Here we selected the negative binomial distribution as the error distribution for count data, which distribution allows one to estimate the variance in addition to the mean. Clearly, the choice of the Poisson distribution would have been too restrictive especially for the Scenarios where the animals were assumed to move in groups. In principle, the choice between the negative binomial and other commonly used alternatives for count data could be assessed by model selection tools. However, in practice we expect that with most data sets, there is only limited information to disentangle among different error distributions.

Although we have illustrated the methods here in the context of track count data, the methodology presented is applicable also for many other kinds of indirect observations, such as dung pile counts or camera trap data. Furthermore, the spatio-temporal smoothing model can be considered as a generic statistical model capturing variation in ecological processes over space and time, and it can be combined with other kinds of observation models than those related to random encounters. The hierarchical nature of the modelling framework allows for many kinds of extensions, such as accounting for variation in movement distances as a function of covariates, or incorporating covariates in the estimation of the spatio-temporal field to improve accuracy and to bring insights on factors generating variation in population density.

## Supporting Information

S1 Appendix Modeling variation in population density as a spatio-temporal and a temporal random effect. (DOCX)

S2 Appendix Numerical integration of population size. (DOCX)

S1 Fig Evaluating the performance of model ST in estimating total population size. Log true and estimated population sizes (gray dots), log-linear regression lines (solid black) and ideal fits (dashed black lines) for each Scenario (A–F). Black square is mean ratio of true and estimated population sizes or centre of gravity of the dots. See main text for description of the Scenarios and the models.
(TIF)

S2 Fig Evaluating the performance of model T1 in estimating total population size. Log true and estimated population sizes (gray dots), log-linear regression lines (solid black) and ideal fits (dashed black lines) for each Scenario (A–F). Black square is mean ratio of true and estimated population sizes or centre of gravity of the dots. See main text for description of the Scenarios and the models.
(TIF)

S3 Fig Evaluating the performance of model T2 in estimating total population size. Log true and estimated population sizes (gray dots), log-linear regression lines (solid black) and ideal fits (dashed black lines) for each Scenario (A–F). Black square is mean ratio of true and estimated population sizes or centre of gravity of the dots. See main text for description of the Scenarios and the models.
(TIF)

S4 Fig Evaluating the performance of model FMP in estimating total population size. Log true and estimated population sizes (gray dots), log-linear regression lines (solid black) and ideal fits (dashed black lines) for each Scenario (A–F). Black square is mean ratio of true and estimated population sizes or centre of gravity of the dots. See main text for description of the Scenarios and the models.
(TIF)

S1 Table Model diagnostics for the Simulation scenarios A–F. Log-linear fits for true and estimated population sizes (*β* = slope, *R*^2^ = goodness-of-fit) for each Scenario (A–F) and model (ST, T1, T2, FMP). Mean ratio of true and estimated population sizes (*α*) and standard deviation (*SD*[*α*]). Percentage of estimates failed (Failed (%)) assumed either too small or too large. See main text for the description of the Scenarios and the models.
(DOCX)
