# Supplementary material for: A Spatio-Temporally Explicit Random Encounter Model for Large-Scale Population Surveys
Source: PLoS One. 2016 Sep 9;11(9):e0162447. doi: 10.1371/journal.pone.0162447 (PMC5017679; doi:10.1371/journal.pone.0162447)
Supplement: S1 Appendix — (DOCX) [file pone.0162447.s001.docx]

# Appendix S1. Modeling variation in population density as a spatio-temporal and a temporal random effect

The unweighted relative density in Eqn 4 is a sum of mean density $\beta$ and structured random variable $z_{it}$. Unlike in the main text, we define $z_{it}=\sum_{k=1}^{V} A_{ikt}\xi_{kt}$ to use approximation to reduce computational cost. The random vector $\boldsymbol{\xi}_{t}$ is estimated at fixed locations $\boldsymbol{v}_{1},\ldots,\boldsymbol{v}_{V}$ (independent of $t$), which are, in general, misaligned with the survey route locations and $V<U$, where $U$ is the number of survey routes. Matrix $\boldsymbol{A}_{t}$ is thus used to map $\boldsymbol{\xi}_{t}$ to the survey routes.

Consecutive years follow the stationary autoregressive process of the first order (AR1), defined by $\xi_{it}=\phi\xi_{i,t-1}+\epsilon_{it}$ for all $t=2,\ldots,T$, where $\left| \phi\right|<1$ describes the degree of temporal dependency and $\epsilon_{kt}$ are spatially structured residuals holding $\boldsymbol{\epsilon}_{t}\sim N(\mathbf{0},\boldsymbol{\Sigma}_{S})$. Elements of the autocovariance matrix $\boldsymbol{\Sigma}_{T}$ are specified by $\mathrm{Cov}\left[ \xi_{it},\xi_{is} \right]=\phi^{\left| s-t \right|}\sigma^{2}/\left( 1-\phi^{2} \right)$ for $1\leq t,s\leq T$ where $\boldsymbol{\Sigma}_{S}\mathbf{=}\sigma^{2}{\tilde{\boldsymbol{\Sigma}}}_{S}$. For the first year, we assumed $\boldsymbol{\xi}_{1}\sim N(\boldsymbol{0},\boldsymbol{\Sigma}_{S}\boldsymbol{/(}1-\phi^{2}\boldsymbol{)})$, which is the stationary distribution of the process, i.e. when $t\to\infty$.

The latent locations $\boldsymbol{v}_{1},\ldots,\boldsymbol{v}_{V}$ are assumed to depend on each other only through Euclidean distances $d_{ij}=\left\| \boldsymbol{v}_{i}-\boldsymbol{v}_{j} \right\|$, hence the spatial process is weakly stationary and isotropic [1]. The spatial autocovariance structure $\boldsymbol{\Sigma}_{S}$ is specified by $\mathrm{Cov}\left[ \epsilon_{it},\epsilon_{jt} \right]=\sigma^{2}$ for all $i=j$ and $\mathrm{Cov}\left[ \epsilon_{it},\epsilon_{jt} \right]=\sigma^{2}C\left( d_{ij},\kappa,\nu\right)$ for all $i\neq j$, where the autocovariance function $C$ is of the Matérn family with an unknown parameter $\kappa>0$ for scale and fixed $\nu=2$ for smoothness [2,3]. However, R-INLA [2] uses the SPDE-approach to approximate the dense spatial autocovariance matrix and therefore $\boldsymbol{\Sigma}_{S}$ is replaced with the approximation. For details, see [2,3].

Since there is no interaction assumed between time and space, i.e. $\mathrm{Cov}\left[ \epsilon_{it},\epsilon_{js} \right]=0$ for all $i$, $j$, $t\neq s$, the full autocovariance matrix for the $VT$-length spatio-temporal random effect $\boldsymbol{\xi=}\left[ \boldsymbol{\xi}_{1}^{⊺}\boldsymbol{\ldots}\boldsymbol{\xi}_{T}^{⊺} \right]^{⊺}$ can be obtained as the Kronecker product $\boldsymbol{\Sigma=}\boldsymbol{\Sigma}_{T}\bigotimes\boldsymbol{\Sigma}_{S}$ [3].

The latent locations are determined based on a triangulation covering the study area and beyond to avoid edge effects. The number of nodes $V$ in the triangulation mesh was chosen to compromise between estimation speed, memory usage and estimate precision [2].

The temporal model in Eqn 5 follows a stationary AR1 process and similarly to the ST model, and we assumed $z_{1}\sim N(0,\sigma^{2}\boldsymbol{/(}1-\phi^{2}\boldsymbol{)})$.

# References

x

| 1. | Cressie N, Wikle C. Statistics for Spatio-Temporal Data: Wiley; 2011. |
| --- | --- |
| 2. | Lindgren F, Rue H. Bayesian Spatial Modelling with R-INLA. Journal of Statistical Software. 2015; 63(19): 1–25. |
| 3. | Cameletti M, Lindgren F, Simpson D, Rue H. Spatio-temporal Modeling of Particulate Matter Concentration through the SPDE Approach. 2013; 97(2): 109–131. |

x
