# Supplementary material for: A Spatio-Temporally Explicit Random Encounter Model for Large-Scale Population Surveys
Source: PLoS One. 2016 Sep 9;11(9):e0162447. doi: 10.1371/journal.pone.0162447 (PMC5017679; doi:10.1371/journal.pone.0162447)
Supplement: S2 Appendix — (DOCX) [file pone.0162447.s002.docx]

# Appendix S2. Numerical integration of population size

Let $\mu_{it}=E[X_{it}]$ be the expected number of tracks crossing the survey route at location$\boldsymbol{s}_{i}$ at year $t$. INLA [1] defines linear predictor $\eta$ related to mean $\mu$ by $\mu(\eta)=eg^{-1}(\eta)$, where $e$ is offset and $g$ is link function for which we used natural logarithm. According to Eqn 4, $\eta_{it}= \log\left( a_{it} \right)$ and $e_{it}=\left. \frac{2}{\pi}M_{i}E[L]D_{it} \right.$. R-INLA [1] provides estimates of the expected population density $\hat{a}_{it}$ at the survey route locations $\boldsymbol{s}_{i}$. We obtained the full density surface estimate by interpolating over $\Omega$ using thin plate smoothing. The estimates $\hat{a}_{it}$ were first square-transformed, interpolated and square-root back-transformed providing values $\hat{a}_{jt}$ at the missing locations indexed by $j$ [2].

The population size integral in Eqn 3 was numerically evaluated as the weighted sum

$$\hat{N}(\Omega,t)\approx\sum_{j=1}^{J} \hat{a}\left( \boldsymbol{s}_{j},t \right)h\left( \boldsymbol{s}_{j} \right)\left| A\left( \boldsymbol{s}_{j} \right)\bigcap\Omega\right|,$$

Eqn S1

where $A\left( \boldsymbol{s}_{j} \right)$ are disjoint sets covering the entire study area $\Omega$. Habitat preferences in Eqn 6 are incorporated into Eqn S1 with $h\left( \boldsymbol{s}_{j} \right)=\left| A\left( \boldsymbol{s}_{j} \right)\bigcap\Omega\right|^{-1}\sum_{k=1}^{K} h_{k}\left| A_{k}\left( \boldsymbol{s}_{j} \right)\bigcap\Omega\right|$, where $\left| A_{k}\left( \boldsymbol{s}_{j} \right) \right|$ is the total area occupied by habitat $k$ within $A\left( \boldsymbol{s}_{j} \right)$.

To estimate credible intervals of $N(\Omega,t)$, we sampled from the joint posterior distribution of the linear predictor $\boldsymbol{\eta}$ 1000 times and transformed each sampled vector $s$ with $\exp\left( \boldsymbol{\eta}_{s} \right)$ to obtain the density estimates. Then we followed the procedure of interpolating and integrating over the study area for each $s$ and $t$ to find the population size as described above. The procedure was repeated for each simulated replicate.

# References

x

| 1. | Lindgren F, Rue H. Bayesian Spatial Modelling with R-INLA. Journal of Statistical Software. 2015; 63(19): 1–25. |
| --- | --- |
| 2. | Hutchinson M. Interpolation of Rainfall Data with Thin Plate Smoothing Splines - Part I: Two Dimensional Smoothing of Data with Short Range Correlation. Journal of Geographic Information and Decision Analysis. 1998; 2(2): 139–151. |

x
