# Supplementary figures and images for: A Spatio-Temporally Explicit Random Encounter Model for Large-Scale Population Surveys
Source: PLoS One. 2016 Sep 9;11(9):e0162447. doi: 10.1371/journal.pone.0162447 (PMC5017679; doi:10.1371/journal.pone.0162447)

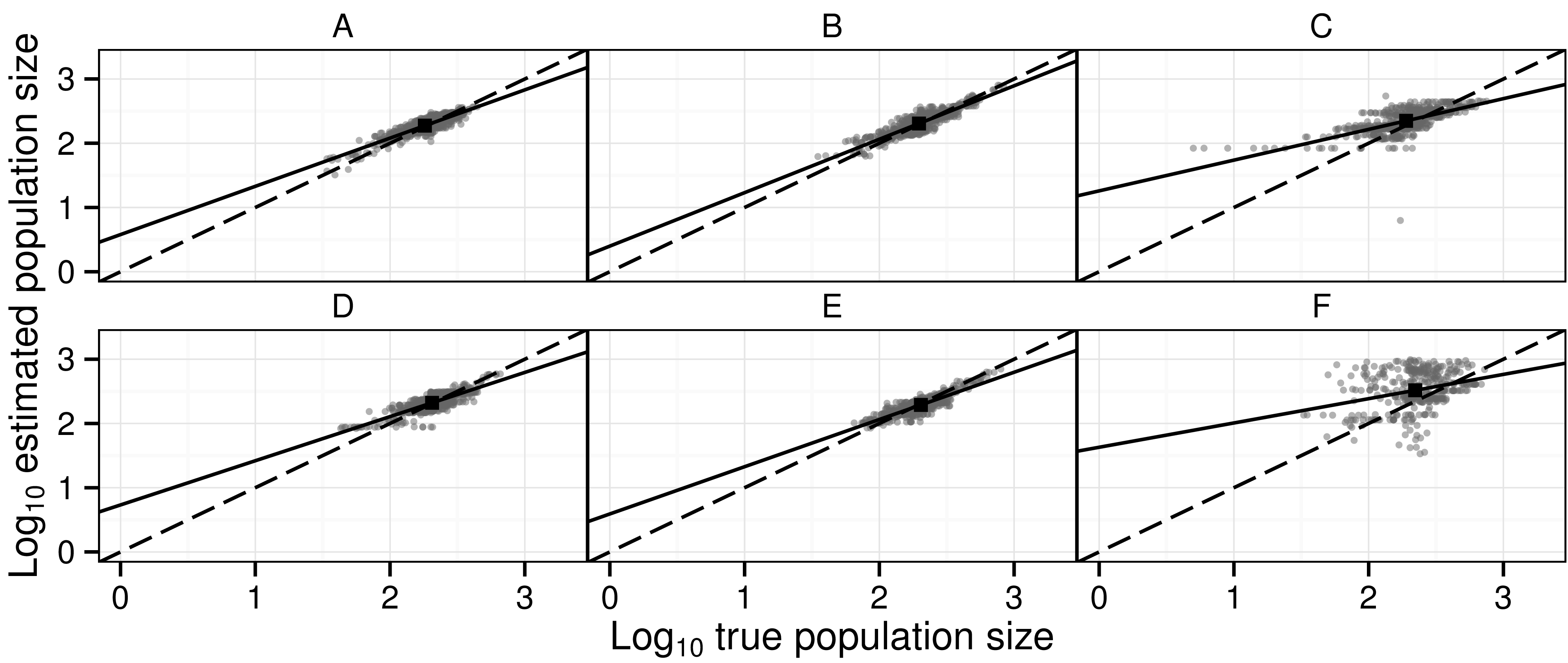

Supplement: S1 Fig — Log true and estimated population sizes (gray dots), log-linear regression lines (solid black) and ideal fits (dashed black lines) for each Scenario (A–F). Black square is mean ratio of true and estimated population sizes or centre of gravity of the dots. See main text for description of the Scenarios and the models. (TIF) [file pone.0162447.s003.tif]

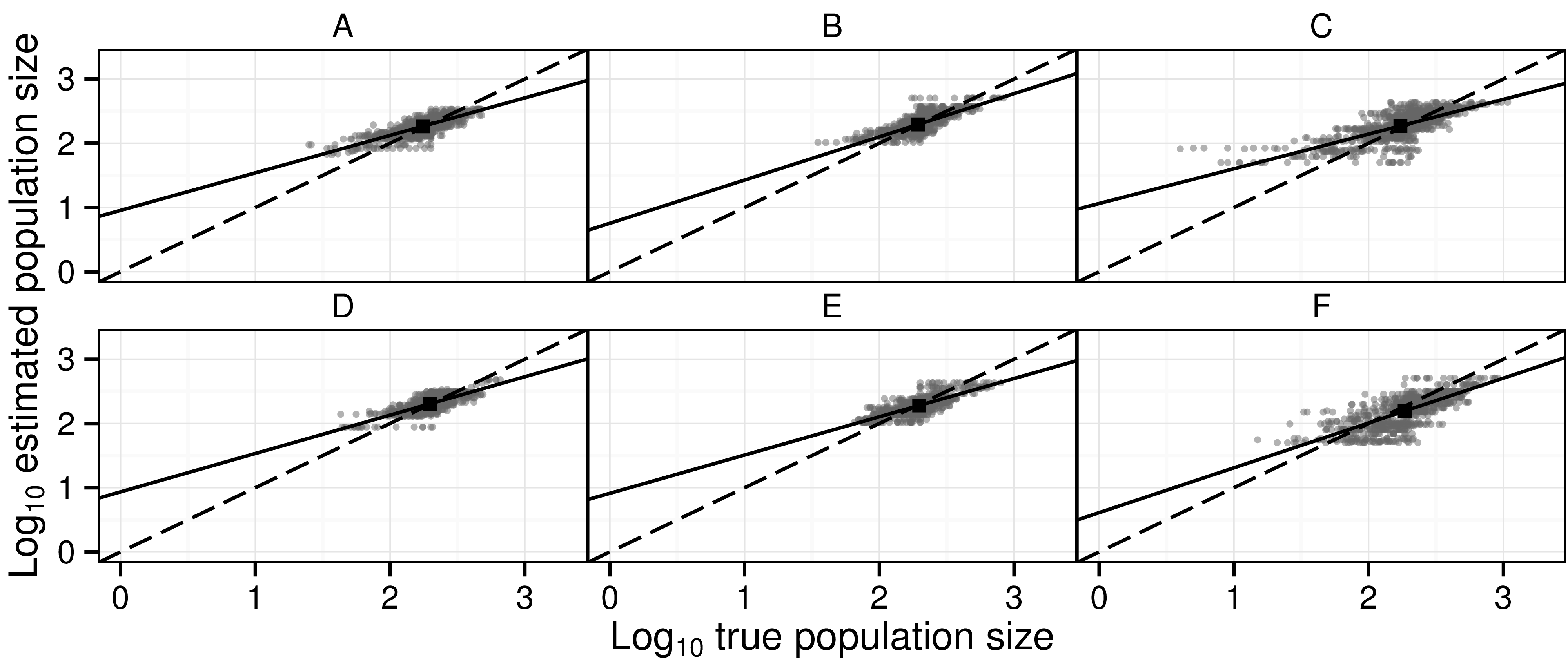

Supplement: S2 Fig — Log true and estimated population sizes (gray dots), log-linear regression lines (solid black) and ideal fits (dashed black lines) for each Scenario (A–F). Black square is mean ratio of true and estimated population sizes or centre of gravity of the dots. See main text for description of the Scenarios and the models. (TIF) [file pone.0162447.s004.tif]

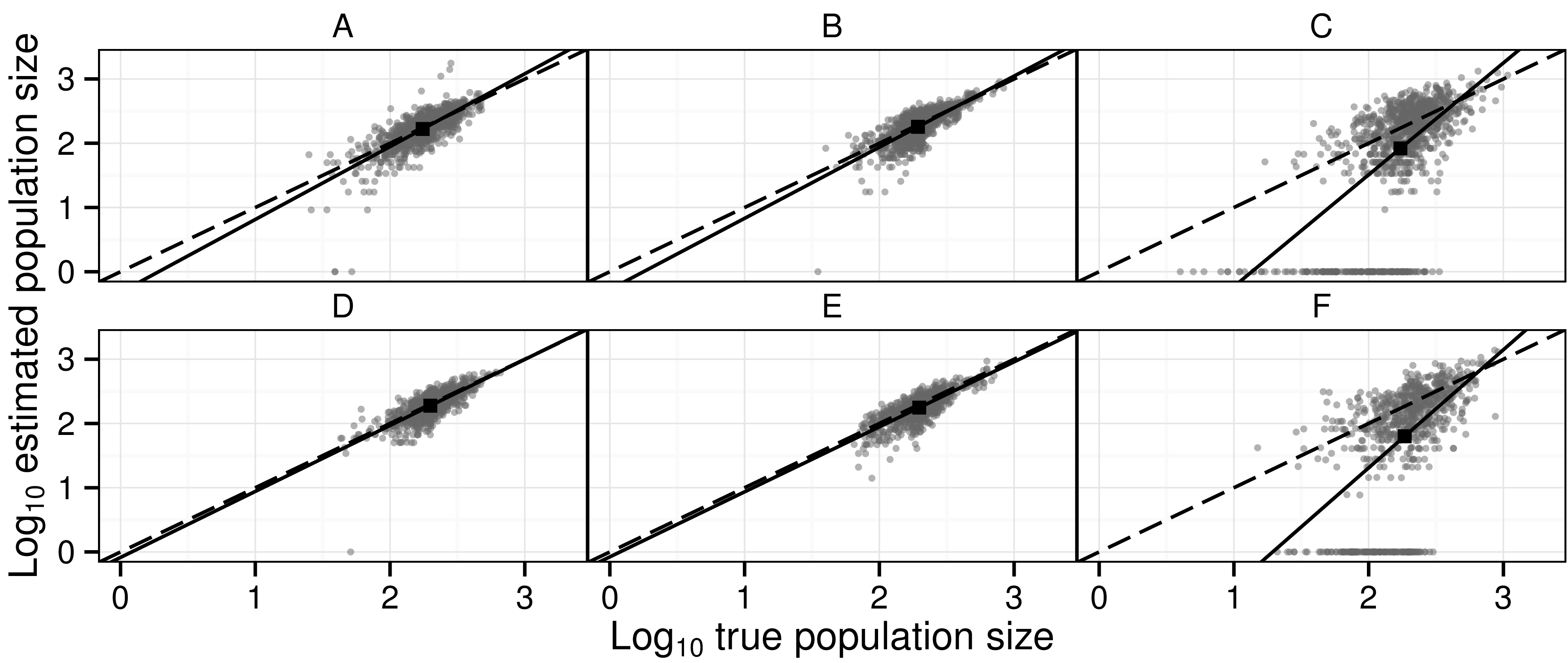

Supplement: S3 Fig — Log true and estimated population sizes (gray dots), log-linear regression lines (solid black) and ideal fits (dashed black lines) for each Scenario (A–F). Black square is mean ratio of true and estimated population sizes or centre of gravity of the dots. See main text for description of the Scenarios and the models. (TIF) [file pone.0162447.s005.tif]

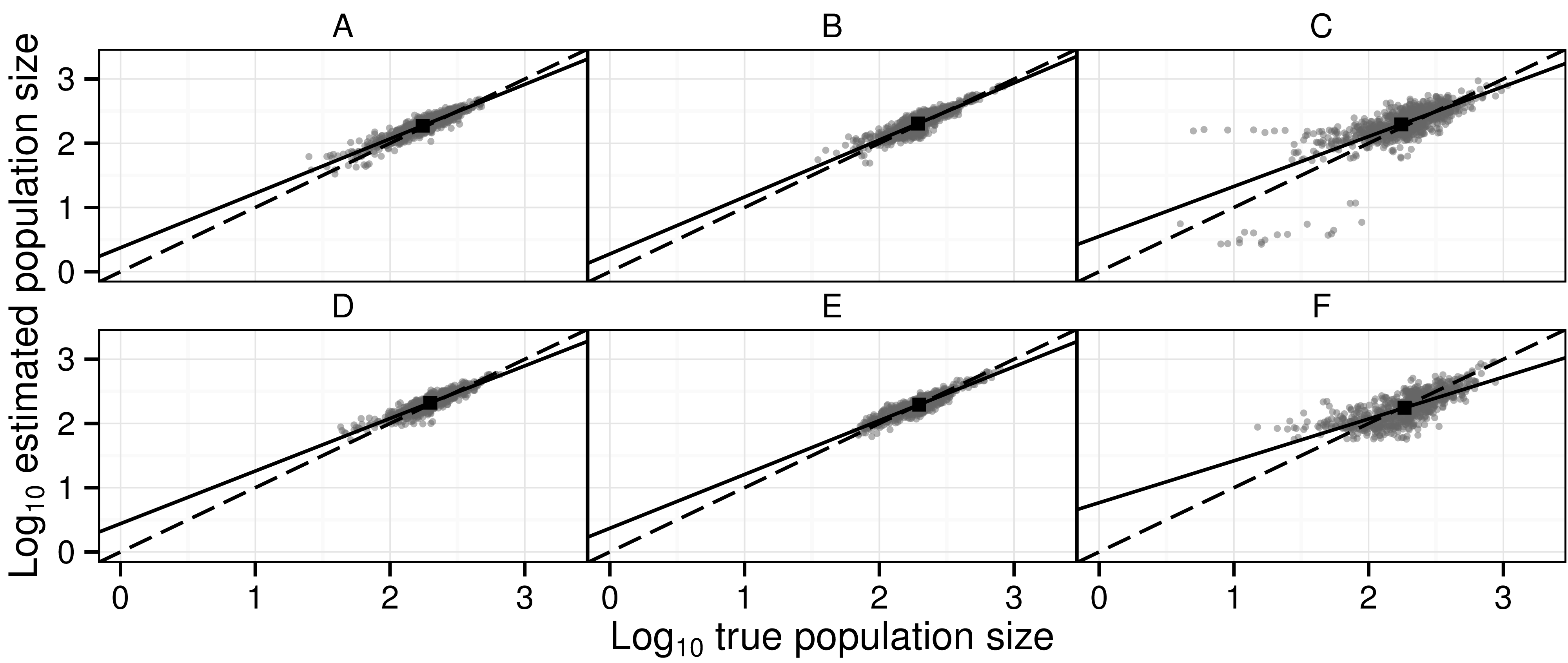

Supplement: S4 Fig — Log true and estimated population sizes (gray dots), log-linear regression lines (solid black) and ideal fits (dashed black lines) for each Scenario (A–F). Black square is mean ratio of true and estimated population sizes or centre of gravity of the dots. See main text for description of the Scenarios and the models. (TIF) [file pone.0162447.s006.tif]
