# Supplementary material for: A Spatio-Temporally Explicit Random Encounter Model for Large-Scale Population Surveys
Source: PLoS One. 2016 Sep 9;11(9):e0162447. doi: 10.1371/journal.pone.0162447 (PMC5017679; doi:10.1371/journal.pone.0162447)
Supplement: S1 Table — Log-linear fits for true and estimated population sizes (β = slope, R2 = goodness-of-fit) for each Scenario (A–F) and model (ST, T1, T2, FMP). Mean ratio of true and estimated population sizes (α) and standard deviation (SD[α]). Percentage of estimates failed (Failed (%)) assumed either too small or too large. See main text for the description of the Scenarios and the models. (DOCX) [file pone.0162447.s007.docx]

| **Scenario** | **Model** | $\boldsymbol{\beta}$ | $\boldsymbol{R}^{\boldsymbol{2}}$ | $\boldsymbol{\alpha}$ | $\mathbf{SD}\boldsymbol{[\alpha]}$ | **Failed (%)** |
| --- | --- | --- | --- | --- | --- | --- |
| A | FMP | 1.13 | 0.58 | -0.01 | 0.07 | 0 |
| A | ST | 0.75 | 0.79 | 0.01 | 0.02 | 24 |
| A | T1 | 0.58 | 0.65 | 0.01 | 0.04 | 0 |
| A | T2 | 0.85 | 0.84 | 0.01 | 0.02 | 0 |
| B | FMP | 1.11 | 0.58 | -0.01 | 0.06 | 0 |
| B | ST | 0.83 | 0.79 | 0.00 | 0.03 | 14 |
| B | T1 | 0.67 | 0.58 | 0.00 | 0.03 | 0 |
| B | T2 | 0.89 | 0.77 | 0.01 | 0.03 | 0 |
| C | FMP | 1.74 | 0.36 | -0.17 | 0.38 | 0 |
| C | ST | 0.48 | 0.45 | 0.02 | 0.06 | 35 |
| C | T1 | 0.54 | 0.54 | 0.01 | 0.07 | 0 |
| C | T2 | 0.78 | 0.55 | 0.01 | 0.08 | 2 |
| D | FMP | 1.03 | 0.58 | -0.01 | 0.05 | 0 |
| D | ST | 0.69 | 0.67 | 0.00 | 0.03 | 16 |
| D | T1 | 0.60 | 0.58 | 0.00 | 0.03 | 0 |
| D | T2 | 0.82 | 0.80 | 0.01 | 0.02 | 0 |
| E | FMP | 1.01 | 0.60 | -0.02 | 0.04 | 0 |
| E | ST | 0.74 | 0.74 | -0.01 | 0.02 | 16 |
| E | T1 | 0.60 | 0.58 | 0.00 | 0.03 | 0 |
| E | T2 | 0.84 | 0.82 | 0.00 | 0.02 | 0 |
| F | FMP | 1.84 | 0.30 | -0.23 | 0.42 | 0 |
| F | ST | 0.38 | 0.09 | 0.05 | 0.10 | 52 |
| F | T1 | 0.70 | 0.54 | -0.02 | 0.06 | 0 |
| F | T2 | 0.65 | 0.55 | -0.01 | 0.06 | 0 |
